# Object-based attentional selection modulates anticipatory alpha oscillations

**DOI:** 10.3389/fnhum.2014.01048

**Published:** 2015-01-12

**Authors:** Balázs Knakker, Béla Weiss, Zoltán Vidnyánszky

**Affiliations:** ^1^Brain Imaging Centre, Research Centre for Natural Sciences, Hungarian Academy of SciencesBudapest, Hungary; ^2^Faculty of Information Technology and Bionics, Pázmány Péter Catholic UniversityBudapest, Hungary; ^3^Department of Cognitive Science, Budapest University of Technology and EconomicsBudapest, Hungary

**Keywords:** object-based attention, EEG, alpha oscillations, faces, words

## Abstract

Visual cortical alpha oscillations are involved in attentional gating of incoming visual information. It has been shown that spatial and feature-based attentional selection result in increased alpha oscillations over the cortical regions representing sensory input originating from the unattended visual field and task-irrelevant visual features, respectively. However, whether attentional gating in the case of object based selection is also associated with alpha oscillations has not been investigated before. Here we measured anticipatory electroencephalography (EEG) alpha oscillations while participants were cued to attend to foveal face or word stimuli, the processing of which is known to have right and left hemispheric lateralization, respectively. The results revealed that in the case of simultaneously displayed, overlapping face and word stimuli, attending to the words led to increased power of parieto-occipital alpha oscillations over the right hemisphere as compared to when faces were attended. This object category-specific modulation of the hemispheric lateralization of anticipatory alpha oscillations was maintained during sustained attentional selection of sequentially presented face and word stimuli. These results imply that in the case of object-based attentional selection—similarly to spatial and feature-based attention—gating of visual information processing might involve visual cortical alpha oscillations.

## Introduction

A remarkable ability of the visual system is that it can deal with the clutter of visual objects in our environment. Given its limited processing capacity, this can only be achieved via attentional selection, that is, assigning priorities to parts of visual information that are relevant according to behavioral goals (Desimone and Duncan, 1995). In neurophysiological terms, this implies that neural processes related to high-priority visual information—attended regions of space, features or objects—should be facilitated (Mangun, 1995; Hillyard et al., 1998; Kastner et al., 1999). Conversely, it can be advantageous to suppress the neural representation of irrelevant items (distractors) (Slotnick et al., 2002, 2003; Vidnyánszky and Sohn, 2005; Gál et al., 2009). These inhibitory processes are especially important for efficient attentional selection when several objects are simultaneously present, which frequently occurs during everyday visual experience (Seidl et al., 2012; Peelen and Kastner, 2014).

A prominent neural signature of attentional distractor suppression is enhanced oscillatory activity in the alpha frequency band (Klimesch et al., 2007; Jensen and Mazaheri, 2010; Foxe and Snyder, 2011), which can be measured in human subjects non-invasively by means of electroencephalography (EEG). It is well-established that during spatial attentional tasks, the representation of the unattended visual space is inhibited through enhanced alpha activity in the corresponding parts of the visual cortex (Worden et al., 2000; Sauseng et al., 2005; Kelly et al., 2006; Thut et al., 2006; Rihs et al., 2007). More recently, it has been shown that this generalizes to feature-based attention: Snyder and Foxe (2010) demonstrated that anticipatory alpha band power increases can be localized more ventrally when the motion of the presented dot field was task-relevant, as compared to more dorsal sources when attending the color of the same dots.

However, when the visual system is faced with the visual clutter of multiple objects, the units of attentional selection are whole objects (O’Craven et al., 1999). On what level of the visual hierarchy object-based selection operates is an outstanding question in recent research. The findings thus far support the assumption that, besides well-established modulations in category-specific areas in the ventral temporal cortex, object-based attention relies on top-down feedback signals biasing the activity of earlier visual areas based on high-level object knowledge (Cohen and Tong, 2013; Davidesco et al., 2013; Baldauf and Desimone, 2014). Electrophysiological studies on the role of alpha oscillations in gating visual object processing have also been conducted, but in these, attended stimuli and distractors were separated either in space or time (e.g., Jokisch and Jensen, 2007; Payne et al., 2013; Payne and Sekuler, 2014; Zumer et al., 2014). However, whether inhibitory processes involving alpha oscillations are associated with object-based attentional selection in the case of simultaneously present visual objects remains an important unresolved question.

To address this question, we designed a paradigm using word, face and composite word-face stimuli. In each trial, either the word or the face component was cued to be attended, the other being task-irrelevant. To maximize the engagement of object-based selection mechanisms and to minimize the involvement of spatial attention, all stimuli were presented foveally at the same location—that is, words were overlaid on faces in the case of composite stimuli. Words and faces are suitable to probe object-based attention because of the well-known, pronounced lateralization of their processing: category-selective neural activity dominantly takes place in the right and left hemisphere in the case of faces and words, respectively (Kanwisher et al., 1997; Cohen et al., 2000). Based on this, we predicted that object-based attention to either category in a compound word-face display will modulate the hemispheric lateralization of visual cortical alpha oscillations. In particular, attending to faces will lead to increased alpha power in the left hemisphere, which is dominantly involved in the processing of word stimuli, whereas attending to words will increase alpha power in the right hemisphere, which is dominant in face processing. We tested these predictions in the case of sustained object-based attentional selection of face or word stimuli, presented sequentially (six stimuli, each presented for 683 ms) within a trial.

## Materials and methods

### Subjects

Twenty healthy young adults participated in this study. All of them had normal or corrected-to-normal vision; none of them had any history of neurological or psychiatric diseases. All participants gave their informed consent prior to starting the experiment, the procedures of which were approved by the Ethical Committee of the Budapest University of Technology and Economics. The data of three participants was discarded because of excessively noisy EEG recordings (less than 50% of the segments were clean, mean ± SEM for retained subjects: 77 ± 3%), and one subject was discarded because of lack of response in more than 15% of the trials (mean ± SEM for retained subjects: 3 ± 0.7%). So, the data from 16 subjects was analyzed (9 female, mean ± SEM age: 21.4 ± 0.3 years).

### Stimuli and procedure

**Figure 1:**
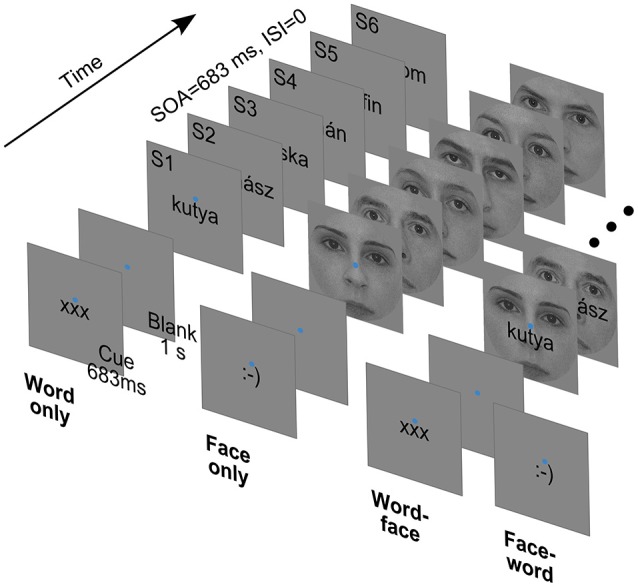
**Cues and sample stimulus trains from the four experimental conditions**. SOA: Stimulus Onset Asynchrony. ISI: Interstimulus Interval.

In the experiment, participants viewed short sequences of word, face and composite word-face stimuli while performing a one-back task (Figure 1).

Face images were front-view grayscale photographs of 13 male and 13 female faces. The 2° × 2° square images were cropped with a circular mask with a diameter of 2° to eliminate external facial features and equated for contrast and luminance. Word stimuli were 26 Hungarian nouns (from two semantic categories: 13 fruits and 13 animals) rendered in black using a 12 point Arial font (maximal vertical extent: 0.4°). Words were 5–7 characters long, corresponding to widths falling between 0.9 to 1.5° of visual angle. From the above, composite stimuli were created by overlaying words centrally on face images (Figure 1, right). All of the stimuli were presented at the center of the screen, slightly (0.2°) above which a blue fixation disc with a diameter of 0.1° was always present. The background was mid-gray, matching the mean luminance of face images.

Trials started with a cue displayed for 683 ms, which was either of the strings “xxx” or “:-)” rendered in the format and position described above. The cue was followed by a blank interval of 1 s, when only the fixation disc was present. Then, six stimuli of one type (word, face or composite) were presented consecutively. Each stimulus was displayed for 683 ms, immediately followed by the next one—there was no interstimulus interval. The intertrial interval, from the offset of the last stimulus to the onset of the next cue, was 2 s long.

Subjects had to deploy their attention and perform the task with respect to either words or faces, as indicated by the cue at the beginning of each trial—“xxx” and “:-)” referring to words and faces, respectively. For each subject, 240 attend-word and 240 attend-face trials were presented in randomized order. In both cases, for a 50% random subset of the trials (120 for attend-word and 120 for attend-face), compound stimuli were used, the non-attended stimulus serving as a distractor. In the remaining trials, only the relevant stimulus was displayed. Thus, there were four experimental conditions (Figure 1): attend-word distractor-absent (*word only*), attend-word distractor-present (*word-face*), attend-face distractor-absent (*face only*) and attend-face distractor-present (*face-word*).

In one third of trials, the sub-category (male vs. female faces, animal vs. fruit words) was alternating throughout the stimulus sequence. In the remaining two thirds of trials, one or two one-back repetitions of stimulus sub-category occurred. The task of the participants was to count these one-back events and indicate how much of them they saw with a three-button mouse after each trial, during the intertrial interval (For example, a “male-female-male-female-male-female” sequence would count as no (zero) one-back repetition, “fruit-animal-fruit-fruit-animal-fruit” would count as one repetition, and so on). This task was designed to sustain the attentional state of subjects throughout the whole trial as much as possible.

Each subject completed 480 trials in 10 runs, leading to 120 trials per condition. Stimuli were presented on a 26” LG LCD monitor at a refresh rate of 60 Hz, viewing distance was 56 cm. Stimulus presentation and subject response registration was implemented in MATLAB 7.1 (The Mathworks Inc., Natick, MA, USA) using PsychToolbox 3 (Brainard, 1997; Pelli, 1997).

### Electrophysiological data acquisition and processing

EEG was acquired using BrainAmp MR amplifiers and an ActiCap system with 62 active electrodes (Brain Products, Munich, Germany) mounted on an elastic cap according to the 10/10 system. An additional lower vertical EOG electrode was placed below the right eye. All channels were referenced to the right mastoid (TP10), the ground was at electrode position AFz. Impedances were kept below 20 kΩ. The sampling rate of EEG was 500 Hz.

Preprocessing and data analysis was done in Brain Vision Analyzer (Brain Products, Munich, Germany) and MATLAB (The Mathworks Inc., Natick, MA, USA) using functions from EEGLAB (Delorme and Makeig, 2004) and custom scripts. The signal was bandpass filtered (Butterworth zero-phase filter in Analyzer, 0.1 Hz-70 Hz, 24 dB/octave). Trial segments containing artifacts were marked using amplitude ([-100 100] µV), amplitude difference (160 µV) and voltage step thresholds (20 µV per sample) and by visual inspection; these segments were not used in further analyses. Surface Laplacian approximation of the scalp current density (SCD) was calculated using the CSD Toolbox (Perrin et al., 1989; Kayser and Tenke, 2006; spline flexibility *m* = 4, *λ* = 10^−5^). SCD-transformed data is reference-free, and is less affected by volume conduction (Nunez and Srinivasan, 2006). Modulations of alpha oscillations was of particular interest in this study, so whole-trial segments were wavelet transformed using a complex Morlet wavelet (MATLAB cwt function, “cmor1-1” wavelet) with center frequencies 8–12 Hz with 0.5 Hz steps. Afterwards, mean log (with base 10) power time series were computed for segments time-locked to each stimulus onset, averaging over trials and frequency bins.

### Statistical analysis

To investigate the modulation of anticipatory alpha oscillations during the stimulus train, mean prestimulus alpha power was extracted from [−50 −200] ms time windows before each stimulus onset from S2 to S6. This window was chosen to minimize the influence of both the previous and the next evoked response, focusing on induced modulations. The main effects of category (attend word vs. attend face), distractor (absent vs. present) and their interaction were first assessed over the whole scalp using cluster-based permutation tests (cluster-forming threshold *p* = 0.05, 999 permutations, adjacent stimulus windows and electrodes in less than 5 cm distance were considered neighbors, hypothesis tests were two-tailed) using functions implemented in FieldTrip (Maris and Oostenveld, 2007; Oostenveld et al., 2011). For the category × distractor interaction, there were no significant clusters (all *p* > 0.1), therefore interaction effects between the main effects were not considered in further analyses. To assess anticipatory attentional modulations before S1, a similar permutation test for the category effect involving only the spatial dimension was performed on a longer pre-S1 time window ([−100 −600] ms before S1 onset).

From the whole-scalp results of the two main effects, electrode pools of interest for further analysis were defined in the following way. First, electrodes where significant differences were consistently present across the whole temporal extent of the cluster (S2–6 for the category effect, S2–4 for the distractor effect, see Section Results) were selected. Second, symmetric hemispheric electrode pools were formed, assuring that the pair of each electrode is included in the contralateral pool (For example, on PO3 the category effect was always sub-threshold, but it was added to the left pool for the category effect as a pair of PO4). The electrode pools acquired this way are highlighted with bold labels on Figures 3, 4. Average power during the five pre-stimulus time windows in these pools was analyzed using two ANOVAs (one for the category effect and one for the distractor effect) to evaluate how the effect differed across hemispheres or throughout the stimulus sequence. In these analyses, the factor “sequence” represented position in the stimulus sequence, and “hemisphere” was used to capture lateralization effects. The interactions of these two factors with the current main effect of interest (category or distractor) were also assessed, but not the main effects themselves, as they were already quantified in the whole-scalp statistics stage.

**Figure 2:**
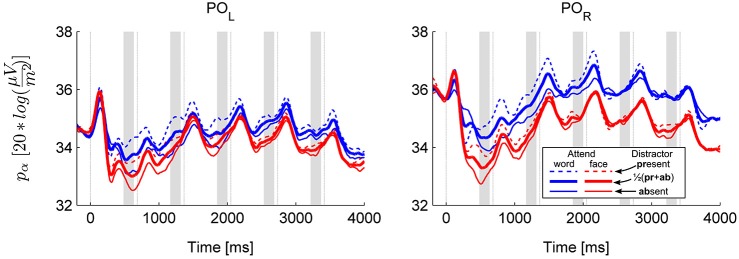
**Grand average alpha power over the left (PO_**L**_: O1 and PO3) and right (PO_**R**_: O2 and PO3) parieto-occipital cluster**. The temporal evolution of alpha power in all four conditions is shown separately (solid and dashed thin lines for distractor absent and present conditions, respectively). In addition, the thick lines show the marginal means for the main effect of category. Thin vertical lines are at the times of stimulus onsets (S1–S6), shaded areas depict pre-stimulus time windows of interest where anticipatory activity was assessed.

**Figure 3:**
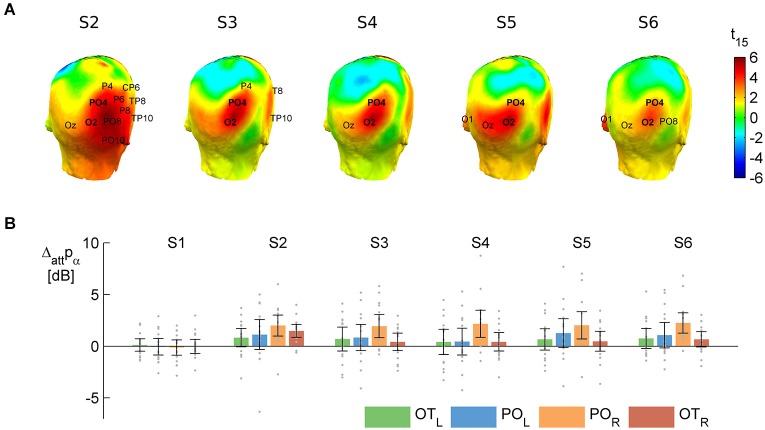
**The main effect of attention to object category on alpha power**. *T*-values **(A)** and raw difference values **(B)** calculated as attend word minus attend face, averaging over distractor absent and distractor present. Positive values indicate larger alpha power to words than faces. **(A)** Results of the cluster-based permutation test on the attentional modulation of anticipatory alpha activity in the prestimulus time windows (see shaded areas on Figure 1) before S2–S6. On the head plots, the color scale shows the results of the parametric *t*-test. The permutation test yielded a significant spatio-temporal cluster—electrodes that are in this cluster in a given pre-stimulus window are marked on the respective head plots. The names of the two electrodes where the effect was consistently significant (O2 and PO4) are in bold. These and their contralateral pairs (O1 and PO3) were pooled and used in further analysis. **(B)** Means (bars) and 95% confidence intervals (error bars) of the attentional difference at the electrode pools selected for further analysis (PO_L_: O1, PO3 and PO_R_: O2, PO4) and two more lateral electrode pools (OT_L_: PO7, P7, PO9 and OT_R_: PO8, P8, PO10). Gray dots mark individual difference scores.

**Figure 4:**
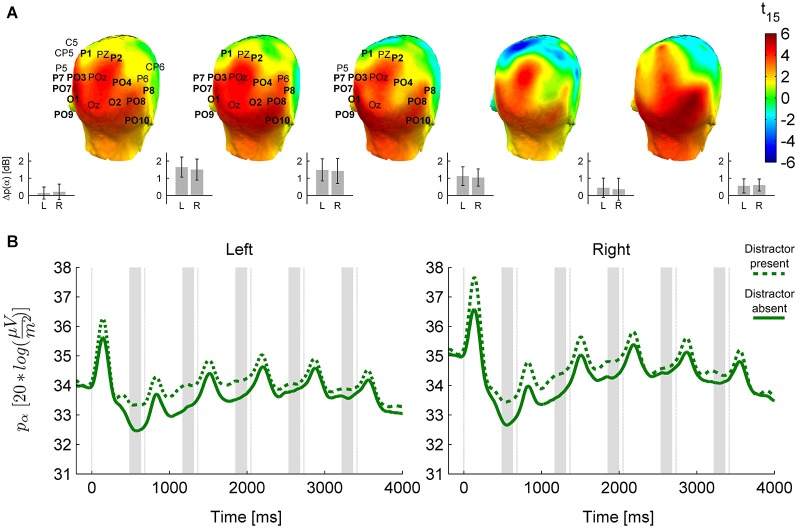
**The distractor effect on alpha power**. **(A)** Head plots for *t*-values of the distractor modulation (distractor present minus absent) of anticipatory alpha activity in prestimulus time windows before S2–S6. As on Figure 3, electrodes marked are in the cluster of significant difference yielded by the permutation test, and bold electrode names are the ones consistently present in the cluster throughout its temporal extent (pre-S2 to pre-S4), which are used in further analysis. “Left” (L) and “Right” (R) on this figure refer to pools of these electrodes from either hemisphere (Left: P7, PO7, PO9, PO3, O1, P1; Right: P8, PO8, PO10, PO4, O2, P2). Insets on the lower right side of each head plot depict means and 95% confidence intervals of the distractor-related difference in the Left and Right pools; on the left of the figure, this is also shown for the pre-S1 interval. **(B)** Temporal evolution of alpha activity in the presence and absence of distractors in the Left and Right pool selected from the distractor-effect cluster.

Task performance was evaluated by comparing accuracies (percentage of correct responses) in all four conditions in a repeated measures ANOVA with factors “category” and “distractor”.

*Post hoc* comparisons were conducted using Tukey’s Honestly Significant Differences procedure. The Huynh-Feldt correction for violation of sphericity was applied where necessary (indicated by ε_H-F_; for the *F*-tests, uncorrected degrees of freedom are reported).

### Eye tracking data acquisition and analysis

Eye movements were recorded using IView X Hi-Speed (SensoMotoric Instruments) at a sampling rate of 240 Hz. Data was cleaned of blinks and detrended, then segmented as described in the ERP processing section. To assess fixation stability, the root mean square deviation from the fixation dot across trials was calculated for each time point and then averaged within each [−200 100] ms peristimulus interval for each condition. Then, these RMS fixation stability values were compared in a repeated measures ANOVA with factors “category”, “distractor” and “sequence”.

## Results

### Behavior

The behavioral results showed that subjects’ accuracy was similar in the attend-face (76 ± 2%, mean ± SEM) and attend-word (77 ± 2%) conditions (main effect of category: *F*_(1, 15)_ = 0.11, *p* = 0.74). The presence of distractors had a significant effect on performance both when faces and words were attended (distractor absent: 79 ± 2%; distractor present: 74 ± 3%; main effect of distractor: *F*_(1,15)_ = 20.43, *p* = 0.00041; category × distractor interaction: *F*_(1,15)_ = 0.00005, *p* = 0.99). These results imply that visual category related attentional effects in the EEG results cannot be accounted for by differences in attentional load or overall task difficulty between the conditions when faces and words were attended.

### Electrophysiology

The results revealed that anticipatory alpha activity measured on parieto-occipital electrodes was modulated depending on whether participants were cued to attend to faces or words (Figure 2, thick lines; Figure 3), regardless of the presence of distractors (Figure 2, solid and dashed thin lines). Alpha power over the right parieto-occipital cortex (Figure 3) was significantly higher when words were attended than when faces were task-relevant (permutation test, cluster-level *p* = 0.02). Importantly, this object category based attentional modulation of alpha power showed a hemispheric lateralization: attending to words as compared to faces led to significantly larger increase in alpha activity over the right than the left hemisphere (follow-up ANOVA on O1,PO3 and O2,PO4 pools: category × hemisphere interaction *F*_(1,15)_ = 6.04; *p* = 0.027).

The object category-dependent attentional effect on anticipatory alpha activity did not arise before S1 (permutation test on time window −100 to −600, cluster-level *p* ≥ 0.1, see also Figure 3B). This is possibly due to our stimuli being long enough to allow post-onset orienting, exerting no time pressure that would require deployment of attention prior to the first stimulus. Before S2, it had a broader topography extending to right temporal electrodes, but afterwards it was confined to the right parieto-occipital region (Figure 3A), where it did not weaken throughout the whole stimulus sequence (follow-up ANOVA on O1,PO3/O2,PO4 pools: sequence × category interaction: *F*_(4,60)_ = 0.42, *p* = 0.72, ε_H-F_ = 0.67, see Figures 3A,B).

The presence of a distractor stimulus also influenced oscillatory power in the alpha band (permutation test for distractor present > absent, cluster-level *p* = 0.002, see Figure 4), but this modulation was distinct from the category effect in several ways. First, no interaction was found between category and distractor (permutation test, cluster-level *p* > 0.1 for all clusters). Second, the distractor effect had a more widespread topography, covering most of the posterior temporal, centro-parietal and occipital cortex (see Figure 4A). Third, the distractor effect, in contrast to the category effect, weakened and disappeared towards the end of the stimulus sequence (follow-up ANOVA on electrode pools highlighted on Figure 4; distractor × sequence interaction: *F*_(4,60)_ = 5.89, *p* = 0.0028, ε_H-F_ = 0.66; *p*_Tukey_ < 0.0005 for the distractor effect in pre-S2 to pre-S4, *p*_Tukey_ > 0.1 for pre-S5 and pre-S6).

It was also found that alpha power displayed a saturation pattern during the trial in all conditions (the trend is visible on Figures 2, 4; main effect of sequence: *F*_(4,60)_ = 8.17, *p* = 0.001, ε_H-F_ = 0.53 for the electrode pools defined by the distractor effect, *F*_(4,60)_ = 7.96, *p* = 0.0022, ε_H-F_ = 0.47 for the electrode pools defined by the category effect; pre-S2 differing from pre-S3–6 *p*_Tukey_ < 0.02, *p*_Tukey_ > 0.5 for the remaining comparisons), which was due to the fact that alpha desynchronization after S1 was prominent but it gradually became weaker or completely disappeared in the case of subsequent stimuli. This modulation of the strength of alpha desynchronization was more pronounced over the right hemisphere (sequence × hemisphere interaction: *F*_(4,60)_ = 3.81, *p* = 0.036, ε_H-F_ = 0.48 in the distractor-effect electrode pools, *F*_(4,60)_ = 2.48, *p* = 0.087, ε_H-F_ = 0.61 for the category-effect electrode pools; pre-S3 vs. pre-S4–6 *p*_Tukey_ ≥ 0.1 over the left hemisphere, but *p*_Tukey_ < 0.001 for pre-S3 vs. pre-S4–5 over the right hemisphere).

### Fixation stability

To assess fixation stability, we measured the subjects’ gaze position during the experiment. Most importantly, 77% of the recorded gaze position data was within a circle with a radius of 0.5°—subjects fixated properly at the stimulus. Mean deviation from the fixation dot was 0.35°, and did not differ across conditions or stimuli (for all effects, *p* > 0.05).

## Discussion

Our results revealed that during sequential presentation of word and face stimuli, the power of parieto-occipital alpha oscillations increased when attending to words, as compared to when faces were attended. This effect was lateralized to the right hemisphere and persisted throughout the stimulus sequence over the parieto-occipital cortex. The presence of a distractor, as assessed by comparing the compound and single stimulus conditions, also modulated alpha oscillations, but did not interact with the object category-based attentional modulation and had distinct temporal and topographical characteristics.

These results show that visual cortical alpha oscillations are associated with object-based attentional selection: attending to the words resulted in larger power of parieto-occipital alpha oscillations over the right hemisphere—which is specialized for face processing (Kanwisher et al., 1997)—as compared to when faces were attended. Thus, these results suggest that object-based attentional selection of task-relevant and suppression of task-irrelevant information might involve alpha-based inhibitory processes, analogously to that found in the case of spatial (Worden et al., 2000; Sauseng et al., 2005; Kelly et al., 2006; Thut et al., 2006; Rihs et al., 2007) and feature-based attention (Snyder and Foxe, 2010). Although one has to be cautious when interpreting topographic features of EEG results because of the limited spatial resolution of the method (Nunez and Srinivasan, 2006), it is notable that the topography of the attentional effect in this study, especially after S2, appears to be similar to spatial attentional modulations described in the literature (Sauseng et al., 2005; Kelly et al., 2006; Thut et al., 2006). This might suggest that the object-based attentional modulation of alpha oscillations might originate from earlier visual areas instead of higher-level, object-selective areas of the ventral temporal cortex. This is in line with extensive previous evidence that object-based attentional effects propagate to early visual areas (Roelfsema et al., 1998; Cohen and Tong, 2013; Davidesco et al., 2013). Importantly, it has also been shown that the hemispheric asymmetry in the neural processing of words and faces in category-specific temporal areas (Kanwisher et al., 1997; Cohen et al., 2002) also affects earlier visual areas, which is reflected in the right and left visual field advantage for processing of words and faces, respectively (see Ellis, 2004; Yovel et al., 2008; Barca et al., 2011). Therefore, a plausible interpretation of our results is that object-based attention gates object-level visual processing by modulating early visual cortical alpha oscillations in a hemisphere-specific way: by decreasing and increasing alpha power in the hemisphere that is specialized for the processing of task-relevant and task-irrelevant objects, respectively. This would be in accordance with the recent results of Zumer et al. (2014) showing that spatial attention routes visual information flow to higher level object-selective cortex via modulating the power of alpha oscillations in the early visual cortex representing the visual field location of the attended and unattended objects, and thus suggest that spatial and object-based attentional selection might share gating mechanisms involving alpha oscillations.

Such an interpretation of our results is also supported by previous studies (Bollimunta et al., 2008; Mo et al., 2011) showing that in macaques performing an attentional task, alpha activity in the inferior temporal cortex (IT) and early visual cortex have distinct functional and physiological properties. First, alpha activity in the macaque IT, as opposed to V2/V4, has a closed-field laminar source configuration, resulting in a weaker signal on the scalp (Bollimunta et al., 2008). Second, also in contrast to V2/V4, increased alpha power in the IT had facilitatory effects both on neural activity (multiunit activity and gamma power) and visual stimulus processing (Bollimunta et al., 2008; Mo et al., 2011). They also speculated that the laminar organization and information flow found in the IT might be well-suited for feedback to earlier visual areas (Bollimunta et al., 2008). According to these data, it is plausible to assume that the alpha modulations found in this study indeed reflect top-down inhibition in the early visual cortex.

The object category-based attentional modulation was only found over the right hemisphere. This was surprising, as we predicted that a similar effect with an opposite sign (attend faces > attend words) would arise over the left hemisphere. This could possibly result from the fact that faces are known to be intrinsically salient, highly effective distractors that gravitate bottom-up attention (Palermo and Rhodes, 2007; Neumann et al., 2011), thus requiring more top-down inhibition than words. Faces also consist of more complex features and covered a larger area in our stimulus display than words—these could also contribute to the greater demands on inhibitory attentional mechanisms when faces needed to be ignored.

Using stimulus sequences instead of single stimuli we could characterize the effect of sustained object-based attentional selection on anticipatory alpha oscillations. After the onset of the stimulus train, the modulation appeared following the early evoked components, during the alpha event-related desynchronization (ERD), and persisted throughout the whole trial. This result is compatible with an alpha modulation with similar temporal dynamics during sustained spatial attention to rapid serial visual presentation of letter sequences (Kelly et al., 2006). Interestingly, independently of this persistent attentional difference, alpha power gradually increased during the course of the trial—it started to increase after the ERD for S1, and the subsequent ERDs were smaller and smaller, leading to a saturation pattern. In terms of the inhibitory account of alpha oscillations (Klimesch et al., 2007; Jensen and Mazaheri, 2010; Foxe and Snyder, 2011)—interpreting the ERD as a release from tonic baseline inhibition (Pfurtscheller and Lopes da Silva, 1999; Klimesch, 2012)—this means that the visual cortex became less excitable during the course of the trial. This is consistent with gradual adaptation or habituation that is expected to occur during sustained stimulation. On the other hand, Rihs et al. (2009, 2007) observed more pronounced attentional event-related synchronization (ERS) in longer anticipation periods, and suggested that this kind of modulation could underlie the maintenance of an attentional set, as opposed to ERD occurring when attention is initially deployed.

Interestingly, there was no significant difference in the category-specific attentional modulation of visual cortical alpha oscillations between the distractor present and absent conditions: attending to words led to significantly higher alpha power over the right hemisphere as compared to when faces were attended both when task relevant stimuli were displayed alone or together with the distractor stimulus. This appears to suggest that it is the reduction of alpha power over the right hemisphere when faces are task relevant that leads to category-specific attentional effects in our study. This is because category-specific attentional modulation would be expected to be stronger in the presence of a distractor if it was driven by the increase of alpha power when faces are task-relevant. However, it is important to note that previous studies investigating visual spatial attentional selection found strong and sustained increase of alpha power over the hemisphere that represent the task-irrelevant visual field even in absence of distractor stimuli (Rihs et al., 2007, 2009). This suggests that visual cortical representation of the unattended part of the visual field is blocked via increased alpha oscillations independently of whether it contains any distractor stimuli. If one assumes that an analogous gating mechanism operates in the case of spatial and object-based attentional selection, this result would imply that attending to words in the current study could lead to increased alpha oscillations over the right hemisphere that is specialized for the processing of faces even when no distracting faces are presented.

Although distractors had no effect on the lateralized category-specific attentional modulation, alpha oscillations were found to be modulated by distractors. This effect had a topography clearly different from that of the object-category attentional effect, with a broader, bilateral spatial distribution. Interestingly, however, the distractor effect on alpha power was not present throughout the whole stimulus sequence, but weakened substantially towards the end of the trial. This suggests that an unattended second stimulus could be distracting to a different degree in the beginning and in the end of the trial. We sought to confirm this by assessing whether the behavioral distractor effect differed depending on the position of a single target in a trial, but did not find any compatible pattern. It could also be argued that the temporal dynamics of the distractor effect might follow the temporal dynamics of overall alpha power changes during the trial, but we could not soundly establish this relationship either. Therefore, clarifying the neural and cognitive processes underlying this effect would require further research.

To conclude, our results provide the first evidence that object-based attention modulates visual cortical alpha oscillations: attending to a word in a compound, foveally displayed word-face image boosted parieto-occipital alpha oscillations over the right hemisphere. This is consistent with attentional gating in early visual areas, with alpha oscillations involved in the selection of attended and suppression of task-irrelevant visual stimulus representations.

## Author’s contributions

Zoltán Vidnyánszky and Balázs Knakker designed the experiment; Balázs Knakker collected data; Balázs Knakker and Béla Weiss analyzed data; Balázs Knakker and Zoltán Vidnyánszky wrote the manuscript.

## Conflict of interest statement

The authors declare that the research was conducted in the absence of any commercial or financial relationships that could be construed as a potential conflict of interest.
